# Characterization of Ring-Like F-Actin Structure as a Mechanical Partner for Spindle Positioning in Mitosis

**DOI:** 10.1371/journal.pone.0102547

**Published:** 2014-10-09

**Authors:** Huan Lu, Qun Zhao, Hao Jiang, Tongge Zhu, Peng Xia, William Seffens, Felix Aikhionbare, Dongmei Wang, Zhen Dou, Xuebiao Yao

**Affiliations:** 1 Anhui Key Laboratory of Cellular Dynamics and Chemical Biology, University of Science and Technology of China, Hefei, Anhui, China; 2 Department of Physiology, Morehouse School of Medicine, Atlanta, Georgia, United States of America; Georgia Regents University, United States of America

## Abstract

Proper spindle positioning and orientation are essential for accurate mitosis which requires dynamic interactions between microtubule and actin filament (F-actin). Although mounting evidence demonstrates the role of F-actin in cortical cytoskeleton dynamics, it remains elusive as to the structure and function of F-actin-based networks in spindle geometry. Here we showed a ring-like F-actin structure surrounding the mitotic spindle which forms since metaphase and maintains in MG132-arrested metaphase HeLa cells. This cytoplasmic F-actin structure is relatively isotropic and less dynamic. Our computational modeling of spindle position process suggests a possible mechanism by which the ring-like F-actin structure can regulate astral microtubule dynamics and thus mitotic spindle orientation. We further demonstrated that inhibiting Plk1, Mps1 or Myosin, and disruption of microtubules or F-actin polymerization perturbs the formation of the ring-like F-actin structure and alters spindle position and symmetric division. These findings reveal a previously unrecognized but important link between mitotic spindle and ring-like F-actin network in accurate mitosis and enables the development of a method to theoretically illustrate the relationship between mitotic spindle and cytoplasmic F-actin.

## Introduction

For the past decades, our understanding on molecular components, assembly and function of mitotic spindle has achieved great advance [1]. Comparing the biochemical mechanism of mitotic spindle, the biophysical mechanism, especially a mechanical force chain stretching across the mitotic cell, remains elusive. This force chain starts in the region of extracellular substrate-cell cortex fringe with adhesion proteins and actin filaments [2], [3], [4], [5]. As the second part of the force chain, astral microtubules stretch from spindle pole to cell cortex. Astral microtubules conduct the pulling force mainly produced by cortical dynein and regulate spindle positioning and orientation [6], [7], [8], [9], [10], [11]. Spindle positioning, orientation and chromosome segregation are also mechanically orchestrated by mutual motion of Myosin and F-actin around spindle pole [12], [13]. Finally, the pulling and pushing force on spindle microtubules is regulated by motor proteins and mitotic signals [14], [15], [16], [17]. This part is involved in the spindle assembly checkpoint (SAC), which precludes anaphase entry until all chromosomes achieve bi-orientation [18], [19], [20].

Microtubules and F-actin are key players of many biological processes including cell division and embryonic morphogenesis [21], [22]. The cooperation between microtubules and F-actin in regulating the second part of the force chain may be one of the most fascinating and significant events. It is required for spindle positioning in yeast [22], [23], [24] and asymmetrical cell division in polarized epithelial cells [25]. It has been shown that mitotic spindle adapts its orientation by sensing the matrix geometry [22]. The statistics on spindle orientation of the HeLa cells cultured in various shaped matrix substantiates the conclusion that the metaphase spindle angle is influenced by the distribution of cortical F-actin [2], [3]. The main opinion on such influence of matrix towards spindle orientation is that the dynein-mediated astral microtubule-cortex interactions provide the pulling force to dynamically regulate mitotic spindle positioning and orientation [8], [9], [18], [26], [27]. Knockdown of some proteins participating in this process such as MISP, results in more randomized spindle angles as the result of uncontrolled spindle orientation [28]. Inhibiting the polymerization of tubulin or actin by the treatment with Nocodazole or Latrunculin B (Lat B) results in metaphase arrest and abnormally rotated spindles [4]. Collectively, these studies suggest a unique role of microtubule-F-actin interaction in spindle positioning and orientation. However, it remains elusive on how the cytoplasmic force rather than the cortical affects the spindle.

One recent work suggests that Myosin 10 and cytoplasmic actin filaments in *Xenopus Laevis* embryos control spindle length and orientation [13]. Persistently stabilized actin filaments may attenuate the connection between astral microtubules and cell cortex [29]. But it becomes controversial that the reported cytoplasmic actin structure revolving around the spindle has a unique organization and motile pattern [30]. Additionally, a ring-like actin structure related to spindle position and symmetric division is found in mouse zygote, but the dynamic of this structure remains unclear [31]. Mathematical model drawing on the experimental data and model of previous works have been established to deal with the calculation of spindle-cytoskeleton dynamics [2], [10], [32].

We inferred that actin filaments, together with Myosin play a pivotal role in the force chain, and inhibiting Myosin would weaken the interactions between mitotic spindle and cytoplasmic actin filament. In our experiment, we used specific chemical compounds to harness the enzymatic activities of kinases such as Plk1 and Mps1, motility of Myosin, and polymerization of F-actin. We observed that a ring-like F-actin structure forms in metaphase cells or maintains in MG132-arrested cells. Then we presented a model raising possible mechanism underlying the function of cytoplasmic actin filaments. We observed that the drugs perturb the formation of the ring-like F-actin structure, coupled with translated spindles and altered symmetric division. In summary, we characterized the formation of the ring-like F-actin structure as a mitotic event and developed a method to theoretically describe the relationships between mitotic spindle and cytoplasmic F-actin.

## Materials and Methods

### Plasmids and antibodies

The cDNA sequence of human Utrophin (RP11-352E13.1) was obtained from NCBI database and the DNA fragment of UtroCH [30] was synthesized by BGI and cloned into pUC118. We sub-cloned UtroCH into pcDNA-mCherry-N via HindIII and BamHI sites and pEGFP-C2 via EcoRI and BamHI sites. A Kozak-sequence was added on the N-terminal of UtroCH to enhance the expression.

Anti-α-tubulin (DM1A, Sigma Chemical), Secondary antibodies such as FITC-conjugated goat-anti-mouse (Pierce) and Rhodamine-Phalloidin (Invitrogen) were obtained commercially.

### Cell culture and transfection

HeLa cells from American Type Culture Collection (Manassas, VA, USA) were cultured as sub-confluent monolayers in DMEM (Invitrogen) with 10% fetal bovine serum (FBS, Hyclone, Logan, UT) and penicillin-streptomycin (100 international units/ml and 100 mg/ml, respectively, GIBCO) at 37°C with 10% CO_2_. The coverslips were pretreated with collagen (Sigma) according to the manufacturer's instructions.

HeLa cells were double blocked by Thymidine at the concentration of 2 mM to synchronize them at G1/S, then washed with PBS three times and cultured in Thymidine-free medium for at least 8 hours to release. After the first release, mCherry-UtroCH and GFP-tubulin were transfected into cells using Lipofectamine 2000 (Invitrogen) in Opti-MEM (Invitrogen) according to the manufacturer's instructions. When the second block was over, cells were released about 8 hr for immunofluorescence and live-cell imaging.

### Drug Treatments

HeLa cells were grown on coverslips in 24-well plates (Corning, New York). For each drug treatment, we prepared 6 wells of cells. After the second release from Thymidine for 8 hours, cells were treated with aliquots of MG132, 10 minutes later, the first well of cells were collected, labeled as “ 0' ” and fixed for appropriate examination. Remaining wells of cells were exposed to DMSO, BI2536, Blebbistatin, Reversine and Lat B. We collected and fixed these cells every 10 minutes in a row. To collect anaphase cells for analysis of symmetric division, drugs were added 8 hours after release from Thymidine without MG132.

Reversine was used at 0.3 µM, Blebbistatin at 100 µM, BI2536 at 100 nM, Lat B at 10 µM, Nocodazole at 100 ng/ml and MG132 at 20 µM.

### Immunofluorescence microscopy, image processing and quantification

Cells were seeded onto sterile, collagen-treated 12-mm coverslips in 24-well plates (Corning Glass Works, Coring, NY) for transfection or drug treatment, HeLa cells grown on coverslips were rinsed for 1 minute with PHEM buffer (100 mM PIPES, 20 mM HEPES, pH 6.9, 5 mM EGTA, 2 mM MgCl_2_ and 4 M glycerol) and permeabilized for 1 minute with PHEM buffer plus 0.1% Triton X-100. And then extracted HeLa cells were fixed in freshly prepared 3.7% formaldehyde in PHEM buffer and rinsed three times in 1× PBS. HeLa cells seeded on coverslips were blocked with 0.05% Tween-20 in 1× PBS (1× TPBS) with 1% bovine serum albumin (Sigma) for 45∼60 minutes. Then they were incubated with primary antibodies in a humidified chamber for 1 hour and with FITC-conjugated secondary antibodies for 1 hour at room temperature. Phalloidin-Texas was incubated with the secondary antibodies. DNA was stained with 4′-6-diamidino-2-phenylindole (DAPI, Sigma). Samples were examined with a DeltaVision wide-field deconvolution microscope (Applied Precision Inc., WA, USA). Z-stacks were taken at an interval of 0.2 µm. Images were deconvoluted using Softworx (Applied Precision). Measurements of intensities were performed using Image-J (http://rsb.info.nih.gov/ij/) software on un-deconvoluted images. Images were also processed by Image-J.

Quantification of the intensity of F-actin in mitotic cells was conducted as previously described [33]. A specific description of the approach is provided in the article. To be consistent with the statistical diagrams in the figures, all quantified experimental data were displayed in Table S1 and Table S2.

### Live cell imaging

Cells were cultured in a glass-bottomed culture dish (MatTek, MA) pretreated with collagen. Then, the cells were transfected with mCherry-UtroCH/GFP- tubulin and double blocked by Thymidine as described previously. About 8 hours after release, DME medium was replaced with CO_2_- independent medium (Invitrogen) containing 10% FBS and 1% glutamine in a sealed chamber at 37°C. FBS was added according to drug property, as Lat B is unstable in medium with FBS. MG132 was added 5 minutes before the addition of drugs when the cells entered metaphase. We acquired each image every 1 minute over a timeframe of 1 hour or 1.5 hours with Delta-Vision Real Time System built on an Olympus IX-70 inverted microscope base. The live-cell images were also processed with Image-J.

## Results

### Identification of a ring-like F-actin structure during mitosis

**Figure 1:**
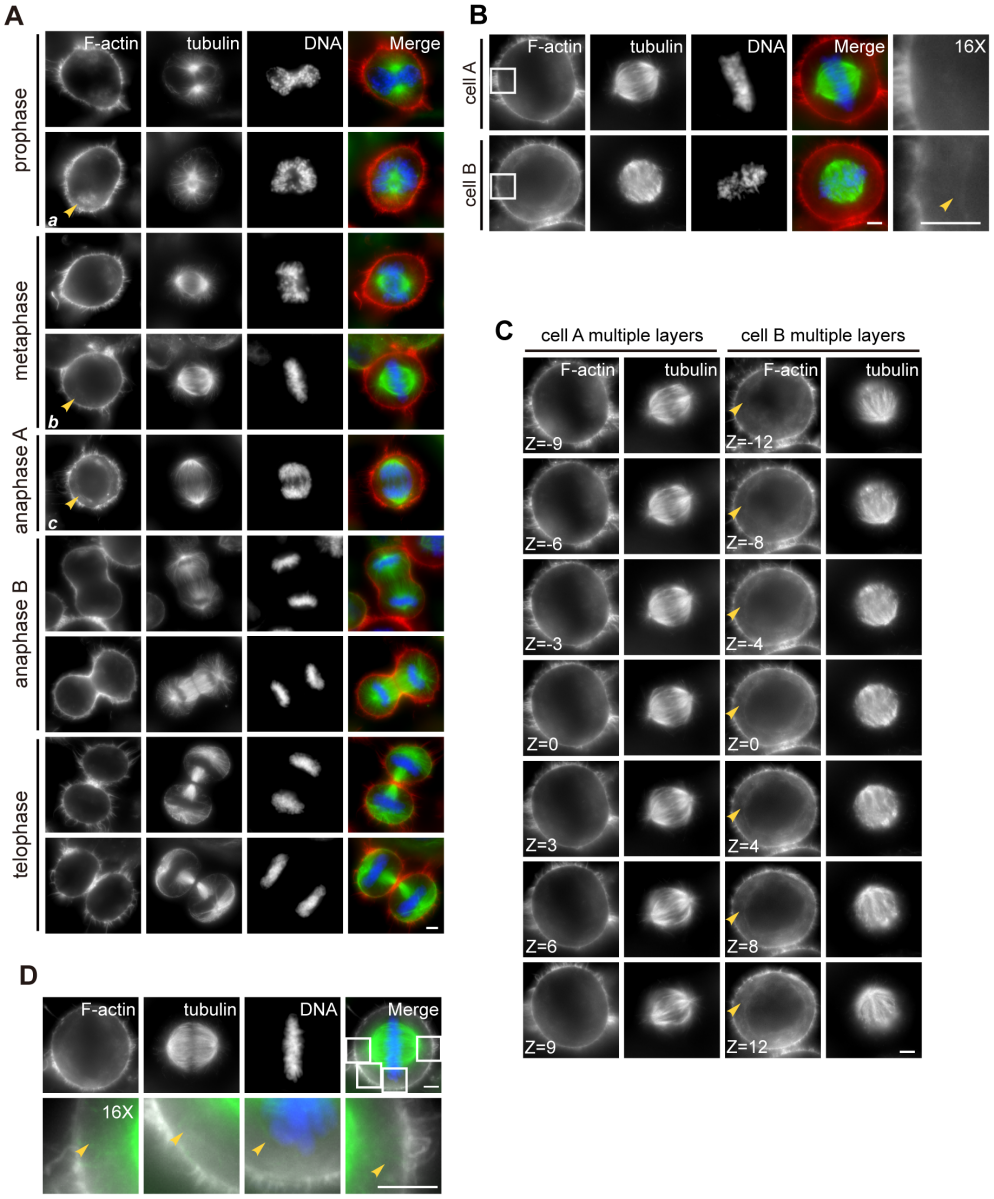
Identification of a ring-like F-actin structure during mitosis. (A) The distribution of F-actin in different phases of mitosis in HeLa cells. Asynchronized cells were fixed and co-stained for F-actin (red), microtubule (green) and DNA (blue). A ring-like F-actin structure appears during metaphase and anaphase and was indicated by the arrows (***a, b, c***). Scale bar, 5 µm. (B) The representative immunofluorescence images of two cells with cytoplasmic F-actin. Cell A represents the cells with slightly rotated spindles, and Cell B represents the cells with distinctly rotated spindle. The boxed areas are shown magnified in the right panels. Scale bar, 5 µm. (C) Multiple-layer images of Cell A and Cell B. The plane passing through the midpoint of spindle poles and parellel to substrate is chosen as Z = 0, and the images in Fig. 1B is taken at the plane Z = 0. The distance between layer Z and layer Z+1 is 0.2 µm. Scale bar, 5 µm. (D) Astral microtubules and cytoplasmic F-actin structure cross and distribute differently in cytoplasm. The yellow arrows indicate the detailed distribution of astral microtubules. The cytoplasmic F-actin structure distributes around the spindle continuously. The boxed areas are shown magnified 16 times in the bottom panels. Scale bar, 5 µm.

It is generally believed that mitotic spindle governs chromosome partitioning and segregation in eukaryotes. However, several lines of recent evidence suggest the role of F-actin-based structure in spindle assembly and positioning [2], [3], [6], [13], [30], [31]. To validate if there are any cytoplasmic F-actin structures in mitotic cells, we carried out immunofluorescence analyses on F-actin and spindle of mitotic cells. To this end, asynchronized HeLa cells were fixed and stained with Rhodamine-Phalloidin and anti-tubulin antibody (DM1A) to visualize F-actin and microtubule, respectively. As shown in Fig. 1A, F-actin staining is mainly distributed on the cortex and retraction fibers through mitosis. Careful examination revealed that the F-actin staining is organized around the spindle poles in prophase cells (Fig. 1A; ***a***, arrow). As the cell goes through metaphase, F-actin staining becomes apparent around the entire spindle (Fig. 1A; ***b***, arrow). In the anaphase A cell, the F-actin staining appears more recognizable (Fig. 1A; ***c***, arrow). Once cells enter into anaphase B, F-actin staining becomes concentrated to contractile ring. During the course of telophase and cytokinesis, the intensity of the F-actin staining in cytoplasm is as low as its intensity in prophase.

**Figure 2:**
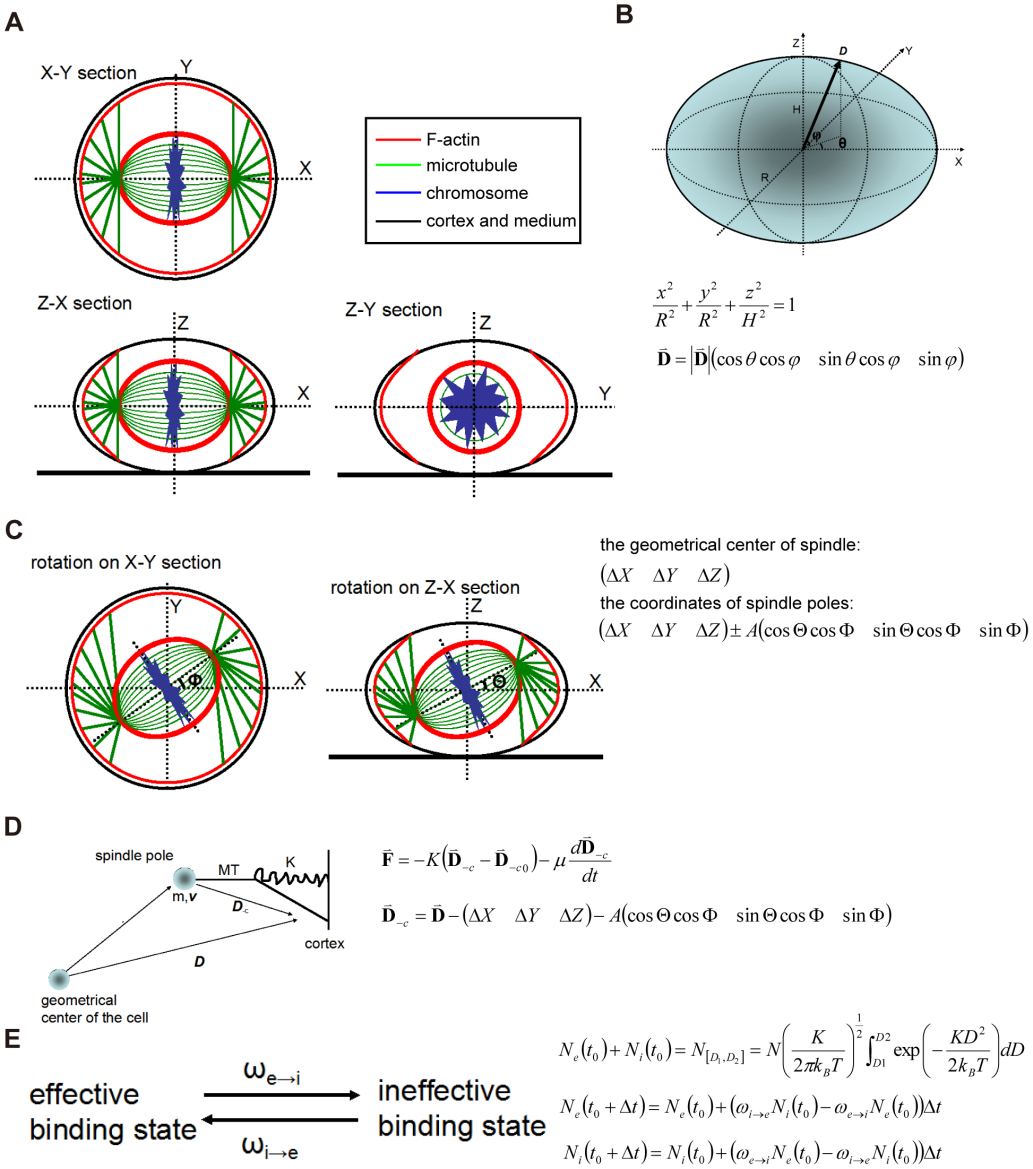
Modeling of spindle positioning and hypothesis of the function of ring-like F-actin structure. (A) The overview of a mitotic cell from three dimensions. X-axis links two spindle poles when the spindle is in its equilibrium position, and Z-axis is vertical to the substrate and passes through the midpoint of spindle poles, and Y-axis is simultaneously vertical to X-axis and Z-axis. Different regions are colored for F-actin (red), microtubule (green), chromosomes (blue), cortex and medium (black). (B) The geometrical hypothesis of cell. A mitotic HeLa cell is supposed to be an ellipsoid with its height H and radius R. Then, the geometry can be described with a formula, and the point on the surface can be positioned with an alterable vector ***D*** and two parameters θ, φ. (C) The schematic diagram of spindle rotation in two sections, X-Y and Z-X. The geometrical center of spindle is supposed to be translated from the geometrical center of cell, and the coordinate of two spindle poles can be expressed with **A**, the distance from spindle pole to equate plate. The rotation in X-Y and Z-X sections can be described with the parameters, **Θ, Φ**. (D) The hypothesis of oscillation. The microtubule-motor complex is simplified as an oscillator, and the oscillation follows Hooke's Law of Elasticity and is influenced by the obstruction from cytoplasm. Here, ***D***_-c_ represents the vector from spindle pole to cell cortex, which can be decomposed into a regular vector ***D*** and the position of one spindle pole. (E) The dynamic transition between effective and ineffective connections or binding state. 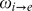 and 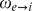 were supposed to be two rate constant for this transition. We also supposed that only the oscillators with its |***D***| in the interval [D_1_, D_2_] and a positive momentum make effective connections. Then the number of effective or ineffective connections can be expressed as displayed in the figure.

The cytoplasmic F-actin is a large collection of bundled actin filaments (Fig. 1B; [34]). It is practically a continuous structure stretching from spindle to cell cortex as well as the astral microtubule. By convention, we defined an X-axis through the spindle poles when spindle is at the equilibrium position, and a Z-axis perpendicular to the imaging plane at the center of the cell. We chose the axis perpendicular simultaneously to X-axis and Z-axis as Y-axis (Fig. 2A). We imaged cells at different layers with an interval of 0.2 µm. The cytoplasmic F-actin changes only slightly along the Z-axis (Fig. 1C, yellow arrows) no matter how much the spindle is rotated. We found that the relative intensity of the cytoplasmic F-actin in different regions varies slightly compared with the Z = 0 plane. Thus we concluded the distribution of cytoplasmic F-actin is isotropic (Fig. S3C, Table for Fig. S3C in Table S1). Compared to the distribution of astral microtubule (Fig. 1D, yellow arrows), the cytoplasmic F-actin structure distributes around the spindle continuously.

To examine this ring-like structure in metaphase arrested cells, an aliquot of HeLa cells was synchronized by a double-Thymidine treatment followed by release into MG132-containing DMEM to prevent anaphase onset. As predicted, treatment of Lat B perturbed the ring-like F-actin structure. These cells treated with other drugs except for Lat B exhibit a distinct actin filament ring (Fig. S3B).

Unlike previous publication [29], F-actin bundling exists during mitosis in normal HeLa cells with considerable intensity. Comparing with the revolving network [30], the cytoplasmic F-actin we found around the mitotic spindle is relatively more relaxed and smooth. The kymograph of the real-time images (Movie S1 and Movie S2) of metaphase arrested cells indicates that the ring-like F-actin structure is relatively less dynamic and cytoplasmic actin filaments have a random motile pattern (Fig. S3E, the teeth appear in a different pattern from reported [30], [44]). Thus, we reasoned that a ring-like cytoplasmic F-actin structure is present around the mitotic spindle in normal HeLa cells during mitosis (Fig. 1C, yellow arrows) and maintains in metaphase arrested cells. This ring-like F-actin staining was mainly observed in metaphase or anaphase of asynchronized cells, and it was observed in all MG132-synchronized cells.

### Modeling of spindle positioning and hypothesis of the function of ring-like F-actin structure

First, we established a 3D-coordinate to localize the point inside the cell. When the spindle is at the equilibrium state during metaphase, the axis vertical to the medium plane is Z-axis, and the axis linking two spindle poles is X-axis, and Y-axis is vertical to X-axis and Z-axis (Fig. 2A). Then we can get an equation to describe the hypothetical ellipsoidal surface of the cell (Fig. 2B), and we used a vector 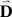 to mark the vector from the center of the cell to the surface. Supposing the translation and rotation simultaneously exist, the distance from spindle pole to equate plate is **A**. The coordinate of the geometrical center of spindle and the rotation angle of spindle in cell coordinate are marked by parameters 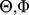 on Z-X and X-Y sections (Fig. 2C).

Then we introduced a 1-D spring oscillator to simulate the motion of microtubule-motor protein complex (Fig. 2D, [35]). Here, 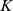 is the elastic modulus, 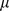 is the frictional coefficient, 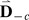 is the vector from one spindle pole to the cortex and 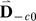 is the similar vector as 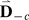 when the oscillator is at the equilibrium position [35].

Supposing all the mass of spindle is concentrated on the spindle pole regardless of the structural dynamics near the equate plate, the force acting on the oscillators is not conservative, and the motion of each spindle pole is independent to each other, we can get the simplified mechanical equation:
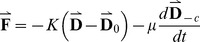
(1)


Then we introduced the offset to the equilibrium position (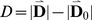), the velocity (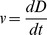) and the momentum (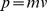, 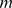 is the equivalent mass of the oscillator). The potential and kinetic energy of each oscillator is:
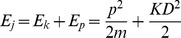
(2)


The distribution function of the oscillators statistically follows an 1-D Maxwell-Boltzmann distribution with its partition function Z, and 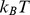 is a modified form of inner energy of the oscillator:
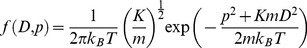
(3)


Supposing the oscillators with D in the interval 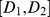 and a positive momentum form effective connections, the number of effective connections is 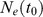 and the number of ineffective connections is 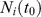 at the initial time point. Moreover, a dynamic transition between 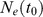 and 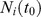 exists during a short period 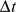, and we supposed the transition rate from 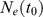 to 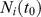 is 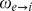 and the transition rate from 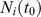 to 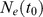 is 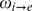 (Fig. 2E). 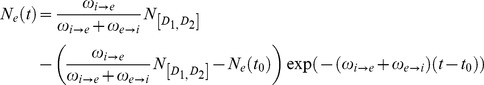
(4)


If we chose 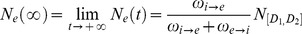 to simplify equation (4), we can get:
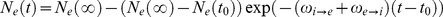
(5)


In conclusion, in a very tiny area on the cell cortex, the approximate total momentum produced by the oscillators is as following, and the pushing force is not considered in this equation:
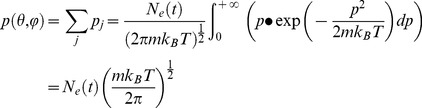
(6)


Supposing the unit vector on the direction of 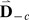 is 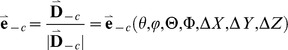, the total momentum produced by all oscillators on the cortex is calculated as following:
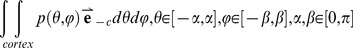
(7)

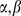
 relate to the distribution of all oscillators.

Then we simplified the calculation of the asymmetric motion. Supposing the coordination of one spindle pole is 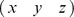, the length of the vector is 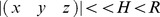, and H is close to R. Then we can get these:
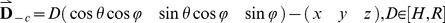
(8)

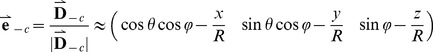
(9)

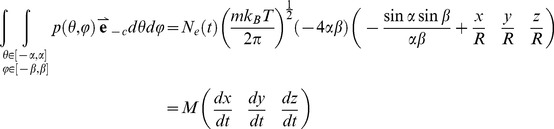
(10)


We concluded that the minute translation of spindle pole will lead to the 3D-oscillation. First, the pattern of the oscillation is based on the components, while **M** is the equivalent mass of the spindle pole. We use a factor 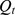 to simplify the presentation of part of the solution:
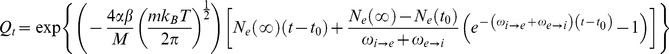
(11)


So 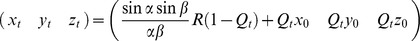 and 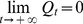
(12)

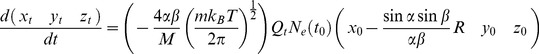
(13)


We concluded that the position of one spindle pole at the final balanced state is


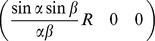
, and the pattern of the oscillation is an exponential decay style. If we choose 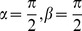 according to the previous works [27], [34], we can get the very significant result: the spindle pole finally stays at the position where the distance from spindle pole to the equate plate is about 0.4 radius of the cell, while spindle is in its balanced place with some oscillation produced by fluctuation of pulling and pushing force [36], [37]. Low concentration of Nocodazole mainly changes the distribution of astral microtubule, which results in altered 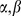 and 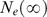.

Then we took the function of ring-like F-actin structure into consideration. We raised three possible manners how the structure performs on the spindle dynamics:

(1) It increases the potential energy of the oscillators. We assumed that the ring-like F-actin structure produces a conserved force field that increases the potential energy of the oscillators. The potential energy is proportional to the momentum of the oscillation and the offset to the equilibrium position. Here the coefficient of proportionality 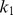 and 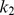 are constants and 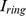 refers to the relative intensity of the ring-like F-actin structure:
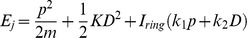



The most probable momentum and offset change from 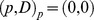 to 
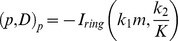
 according to 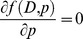 and 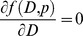. The shortened astral microtubule in metaphase is observed in previous work [29]. Eventually, 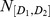 and 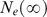 decrease regardless of the metaphase-anaphase transition. 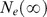, mostly contributing to the factor 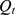, decreases mathematically and results in a decelerated velocity of spindle pole moving from the start point to the equilibrium position. This possibility does not affect the final position.

(2) It decreases the transition rate 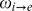 and increases the transition rate 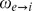. Some works have discovered the phenomenon that the number of astral microtubules raises from metaphase to anaphase (Fig. 1A; [28]). The ring-like F-actin structure is probably a mechanism to control the orchestrated process to reduce the efficient connections between motor protein and cortex. This possibility will also result in a decreased 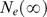.

On the other hand, relevant phenomenon is observed in the cells treated with Lat B. Lat B destroys cortical F-actin more or less and reduces the efficient connections [4].

(3) It increases the viscous force that the cytoplasm performs on the spindle to decelerate the rate of the translation and rotation of the spindle. This possibility is apparently related to the former two reasons. It slows down the velocity of the spindle motion directly, and finally prolongs the time needed to get to the equilibrium position for the cell, which can be analogized to the dynamic process of KT-MT connection. It is also possibly orchestrated by SAC. Moreover, the viscous force generated by the ring-like F-actin structure of proper intensity may significantly reduce the influence by the fluctuation of momentum and precisely position the spindle eventually.

### Perturbation of ring-like F-actin structure formation by chemical inhibitors

**Figure 3:**
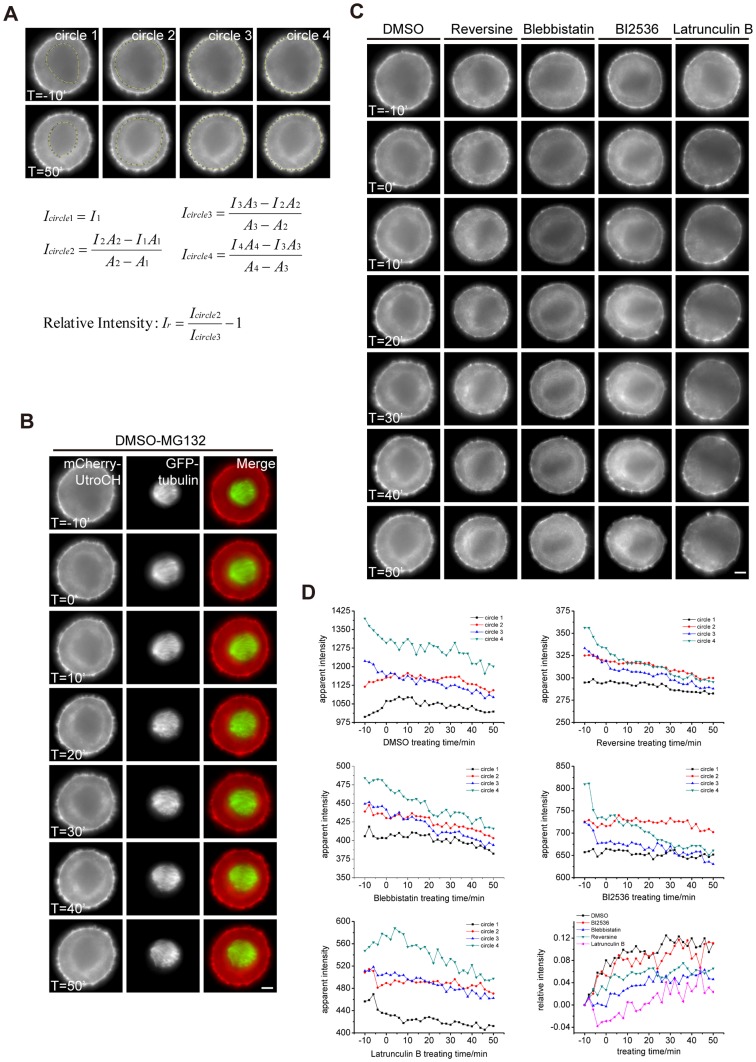
The perturbation of ring-like F-actin structure formation by chemical inhibitors and real-time imaging analyses. (A) The method to measure the apparent intensity of ring-like F-actin structure. A cell is classified into four regions named circle 1, 2, 3 and 4. Circle 1 includes the region of spindle-chromosome complex; circle 2 includes the region of ring-like F-actin structure just around the spindle, excluding the region inside circle 1; circle 3 includes the region of all cytoplasmic F-actin just inside the cortex, excluding the region inside circle 2; circle 4 includes the region of the whole cell body, excluding the region inside circle 3. The formulae to calculate apparent intensity are displayed here. (B) Representative images of a HeLa cell treated with DMSO and MG132. Cells were double blocked and released. MG132 was added at time point -5′, and DMSO was added at 0′. We acquired each image every 1 minute since -10', and the total time was 1 hour. Cells were transfected with GFP-tubulin (green) and mCherry-UtroCH (red) to label microtubules and F-actin, respectively. Scale bar, 5 µm. (C) Representative images of HeLa cells treated with DMSO, BI2536, Blebbistatin, Reversine and Lat B. We used the same method as (B). The influence of chemical treatment on the ring-like structure and spindle position can be observed and measured from the supplemental movies. Scale bar, 5 µm. (D) The apparent intensity of the ring-like structure in each cell measured from the living cell imaging. The curve of circle 3 represents the photo-bleaching along the time. We chose the apparent intensity of circle 3 as the normalization factor. We also chose the apparent intensity of circle 2 as the apprent intensity of ring-like F-actin structure.

Having established a mathematical model to elaborate the probable functions of ring-like F-actin structure in spindle positioning, we sought to define some possible factors involved in its formation. We proposed the following method to calculate the relative intensity of this ring-like F-actin structure in each cell (Fig. 3A). A cell is classified into four regions named circle 1, 2, 3 and 4. Circle 1 includes the region of spindle-chromosome complex; circle 2 includes the region of ring-like F-actin structure just around the spindle, excluding the region inside circle 1; circle 3 includes the region of all cytoplasmic F-actin just inside the cortex, excluding the region inside circle 2; circle 4 includes the region of the whole cell body, excluding the region inside circle 3.

We measured the apparent intensity of F-actin by using ImageJ to draw a polygon along the edge of the circles (Fig. 3A). Then we measured the mean gray-scale value and area inside the circles which are symbolized as following:
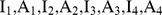



The mean apparent intensity of F-actin within each circle is calculated as following:
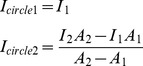


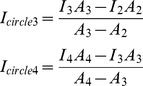



To discover the influence of those drugs on the mCherry-UtroCH labeled F-actin structure, we examined living cell images of each drug-treated group and measured the intensity. To enrich metaphase arrested cells, we added MG132 at -5' and add DMSO, Reversine, Blebbistatin, BI2536 and Lat B at 0', respectively. We found that the ring-like structure formed (Fig. 3B, Fig. S1A, Fig. S1B, Fig. S1C, Fig. S1D, Movie S3–S7). We also found that the intensity of ring-like F-actin structure of all groups, except for the Lat B group, was enhanced to various degrees (Fig. 3C).

To quantify differences between these groups, we measured the relative intensity of the ring-like structure of each group from the living cell images. Then we plotted the time-intensity curves of the four circles and found the slope ΔI_circle3_/Δt relatively unchanged in the experiments (Fig. 3D, Table for Fig. 3D in Table S1), which means I_circle3_ is a reasonable control that changes only with photo-bleaching along time, and it is dynamically stable and changes slightly along Z-axis. So we calculated the relative intensity of the actin structure in the cell as following:
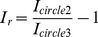



The curve of I_circle2_ represents the formation process of ring-like F-actin structure, and we found that it decreases slightly in each drug-treated group. I_circle1_ keeps nearly a constant deviation to I_circle2_ in each group, while I_circle4_ changes considerably in BI2536, Reversine and Latrunculin B groups. We concluded that the treatment of these drugs affects the cell cortex to some extent. In addition, curves of the relative intensity for each drug-treatment group are plotted together based on the same relative intensity start point. The groups of Blebbistatin, BI2536 and Reversine have curves of ΔI_r_/Δt lying between the groups of DMSO and Lat B (Fig. 3D).

**Figure 4:**
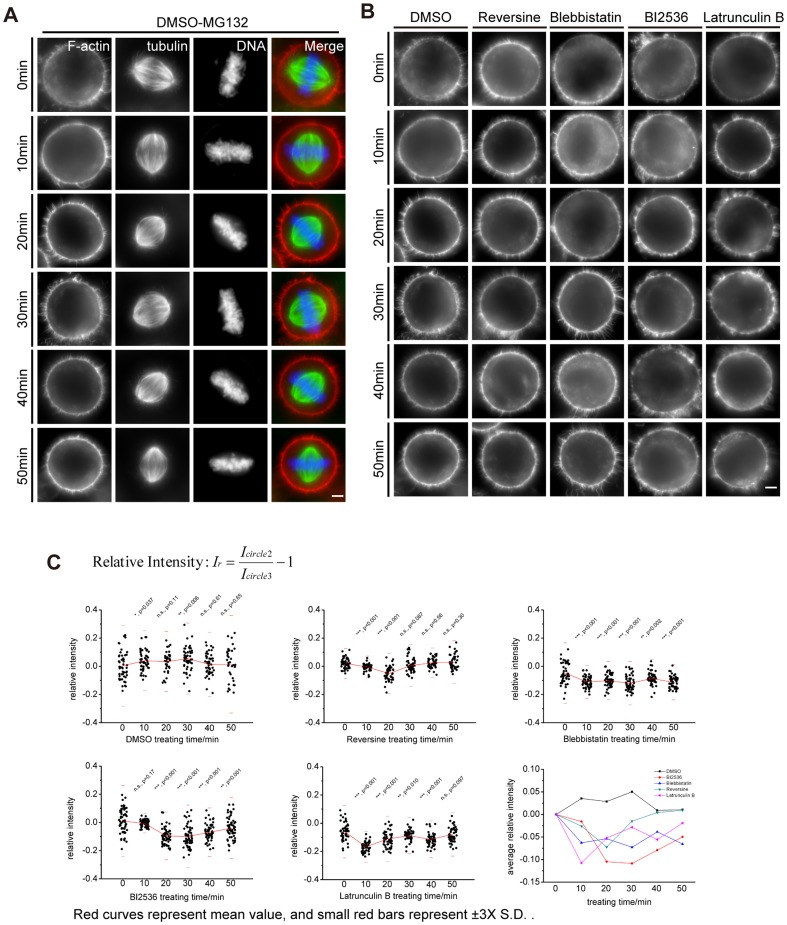
The perturbation of ring-like F-actin structure formation by chemical inhibitors and immunofluorescence analyses. (A) The immunofluorescence images of DMSO treated cells. Drug treatment was performed as mentioned in materials and methods, and cells were collected and fixed after drug treatment, respectively. Cells were immuno-stained for F-actin (red), microtubule (green) and DNA (blue). Scale bar, 5 µm. (B) Presentative immunofluorescence images of HeLa cells treated with DMSO, BI2536, Blebbistatin, Reversine and Lat B. Here we used the same method as (A). Scale bar, 5 µm. (C) The results of the treatment with DMSO, BI2536, Blebbistatin, Reversine and Lat B. MG132 was added at T = −10'. The start points are normalized in the last chart. The last chart suggests that Lat B, BI2536, Blebbistatin and Reversine inhibit the enhancement of the relative intensity. The significances displayed here demonstrate the differences between subsequent time points and T = 0' by t-test. Red curves represent the mean value of the relative intensity of different time points, and red small bars represent the ±3×SD for a 95% confidence interval. Small black points represent the values of relative intensity of each cell. The formula to calculate relative intensity is displayed here.

We next validated the aforementioned results by immunofluorescence of cells taken from given time points (Fig. 4A-DMSO, Fig. S2A-Reversine, Fig. S2B-Blebbistatin, Fig. S2C-BI2536, Fig. S2D-Lat B). We examined images of 30∼50 cells for each time point and measured the intensity of ring-like F-actin structure. DMSO group displays the largest variance in the relative intensity, but the mean is almost at the same level (Fig. 4C, Table for Fig. 4C in Table S1). Except for Blebbistatin group, the other groups have a decline of relative intensity after addition of drugs, but the decline is gradually recovered in a time-dependent manner. The cells do not recover from the treatment of Blebbistatin. These results indicated that the inhibition of Plk1, Mps1 or non-muscular Myosin delays the formation of the F-actin structure, which is consistent with previous works [13], [34], and suggested that some factors link mitotic kinase machinery to the formation of ring-like F-actin structure in mitosis.

The cells arrested in metaphase for 2 hours form this ring-like structure (Fig. S3B). By the way, the ring-like structure still exists in the cells not treated with MG132 (Fig. 1A). Lat B significantly decreases the relative intensity of the ring-like F-actin structure, compared with DMSO group (Fig. S3D, Tables for Fig. S3D and S3F in Table S1). The relative intensity of the ring-like F-actin structures in other groups, except for Lat B and Reversine groups, is significantly enhanced after the metaphase arrest. Treatment with Reversine induces anaphase entry, suggesting that the formation of the ring-like structure is associated with SAC or metaphase-anaphase transition, while Plk1 also participates in the transition. On the other hand, deconstruction of astral microtubule or loss of motion power of Myosin can lead to a more apparent ring-like structure, suggesting potential relations between microtubule, actin filaments and Myosin in the formation of the ring-like structure.

### Treatment of chemical compounds alters spindle position and symmetric division

**Figure 5:**
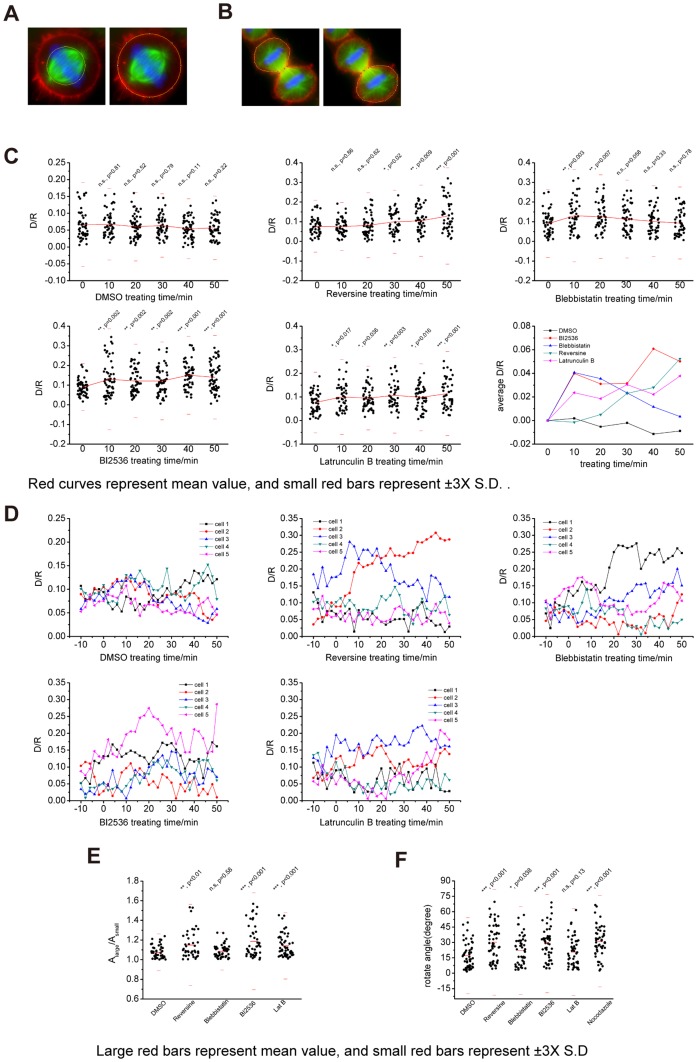
Treatment of chemical compounds alters spindle position and symmetric division. (A) Measurements for the relative translation of the spindle from the cell centroid (D/R). We draw two circles to determine the centroids of spindle and cell on the Z = 0 plane. The first circle highlights the spindle while the other one outlines the cell. Coordinates of the centroids of the circles were used to calculate the offset distance between them. Dividing the distance by the radius of the cell gives the relative distance which is expressed as D/R ratio. (B)Measurements for the area ratio of the daughter cells. The two polygons go around each daughter cell. Then the area ration equals the larger area divided by the smaller area. (C) The D/R changes along time with the treatment of drugs. The method is mentioned in materials and methods. The spindle moved away from the cell centroid in BI2536, Reversine and Lat B groups. However, the shift was finally recovered in Blebbistatin group. The significances displayed here demonstrate the differences between subsequent time points and T = 0' by t-test. Red curves represent the mean value of D/R, and small red bars represent the ±3xSD for a 95% confidence interval. Small black points represent the values of D/R of each cell. (D) The curves presenting D/R along time by living cell imaging. The variation of D/R is enhanced in experimental groups comparing to the DMSO group. (E) The area ratio of daughter cells. BI2536 (p<0.001), Reversine (p<0.01) and Latruculin B (p<0.001) groups are significantly different from DMSO group, which means that the daughter cells of these groups intend to divide asymmetrically, but the Blebbistatin group is not (t-test p = 0.58 and F-test p = 0.94). Large red bars represent the mean values, and short red bars represent ±3xSD for a 95% confidence interval. Small black points represent the values of area ratio of each cell. (F)The rotating angle of spindles in cells treated with drugs. Reversine, BI2536 and Nocodazole groups are significantly different from DMSO group by t-test, which suggests that Mps1 and Plk1 play a role in spindle orientation. The cells were treated with MG132 for 2 hours and then the drugs for 1 hour, respectively.

The reported participation of F-actin structure in the regulation of spindle positioning is the precondition of contractile ring formation and symmetric division [10], [32]. To explore the consequence of attenuated formation of the ring-like structure by the treatment of drugs, we measured the spindle position with the same groups of cells in Fig. 3 and Fig. 4 and cell area ratio of daughter cells in anaphase (Fig. 5A and 5B).

To measure the spindle position (Fig. 5A), we drew two circles to determine the centroid of spindle or cell on the Z = 0 plane. The first circle highlights the spindle while the other one outlines the cell. Coordinates of the centroids of the circles are used to calculate the offset distance between them. Dividing the distance by the radius of the cell gives the relative distance which is expressed as D/R ratio. From the viewpoint of the model, the curves of D/R along time represent the average motion of two spindle poles regardless of the dynamics in midzone. To measure the cell area ratio of daughter cells in anaphase (Fig. 5B), we drew two polygons to measure the area of each cell and the ratio equals the larger area divided by the smaller area. The image is projected on Z axis according to the maximum intensity.

We measured the groups treated with these drugs and made time course curves of spindle position (Fig. 5C, Tables for Fig. 5C and 5D in Table S1). Comparing with relative intensity of ring-like F-actin structure of each group displayed in Fig. 3D, the spindle position of the DMSO group is stable along time, while the spindles of the groups treated with BI2536, Reversine and Lat B are getting further away from the center of cell over time. The spindle position of the Blebbistatin group shows a slight recovery over the same period. Considering the relative intensity, the delay in the formation of ring-like F-actin structure is correlated with the perturbation of spindle position, which is probably due to less obstruction in cytoplasm. The inhibition of Myosin by Blebbistatin delays accurate spindle positioning, suggesting another pathway regulating spindle positioning. Together, these results indicated that the mitotic kinases such as Plk1 and Mps1 and motile Myosin are essential for accurate spindle positioning, which is coupled with the formation of the ring-like F-actin structure.

To assess how the drug treatment influences spindle position in a single cell over time, we examined the spindle position by living cell images of each group (Fig. 5D, Tables for Fig. 5C and 5D in Table S1). We found that the spindle positions in the cells treated with drugs except for DMSO have higher variance than the spindle position in DMSO group.

Both the formation of ring-like F-actin structure and the spindle position in Lat B group are perturbed. However, we should not attribute the perturbed positioning totally to the perturbed formation of ring-like F-actin structure, because the cortical F-actin is also influenced by the drug. Low concentration of Latrunculin A, a similar drug as Latrunculin B, is able to destroy cytoplasmic F-actin, but the completeness of cortical F-actin is conserved [31]. Thus we concluded that the formation of ring-like F-actin structure and spindle positioning are two parallel events under the regulation of mitotic signals and Myosin.

To discover the influence of the drugs on symmetric division, we measured the cell area ratio of daughter cells in anaphase (Fig. 5E, Tables for Fig. 5E and 5F in Table S1). Compared with DMSO group, the area ratios of BI2536, Reversine and Latrunculin B groups are significantly different. However, Blebbistatin group has no significant difference. This may due to the stable spindle position of the cells treated with Blebbistatin (e.g., Fig. S3A). Although there are much more factors involved in the positioning of contractile ring, this ratio also suggests potential relations between the formation of ring-like structure and symmetric division. But the detailed pathway remains unclear.

To address how the ring-like actin structure influences the spindle orientation, we measured the spindle angle and length of metaphase arrested cells (Fig. 5F, Tables for Fig. 5E and 5F in Table S1, Fig. S3B, Fig. S3D, Fig. S3F are same groups of cells). Lat B and Blebbistatin groups do not have significant difference with DMSO group at the level of p<0.01, but Reversine, BI2536 and Nocodazole groups do. Spindles in BI2536 and Nocodazole groups are significantly shorter than control (Fig. S3F, Tables for Fig. S3D and S3F in Table S1), while the spindle length in Reversine, Blebbistatin and Lat B groups has no significant difference from the control. These results suggest that spindle orientation is not significantly influenced by inhibiting Myosin motility or depolymerization of actin filament. The experimental concentration of Lat B may have very limited influence on cortical F-actin to alter spindle orientation in most cells we measured, which is consistent with previous publication [31]. However, altered spindle orientation partially reflects the situation of cortical F-actin. Thus these are not crucial evidences to correlate ring-like structure formation with spindle position.

Taken together, it remains unclear how these drugs influence the cortical stabilization. It is accepted that cortical complex influence spindle oscillation and positioning [10], [11]. On the other hand, spindle orientation is influenced by microtubule-cortex interaction. Thus, both the situations are related to the completeness of cortical complex. Lat B and Blebbistatin mainly affect cytoplasmic F-actin and more or less influence the function of cortical complex. The treatment of Blebbistatin and Lat B have major influence on the formation of the ring-like F-actin structure but have minor influence on cortical complex. On the contrary, Reversine and BI2536 treatment have major influence on cortical complex.

### 3D projected images of a single HeLa cell and 3D spindle positioning analysis

**Figure 6:**
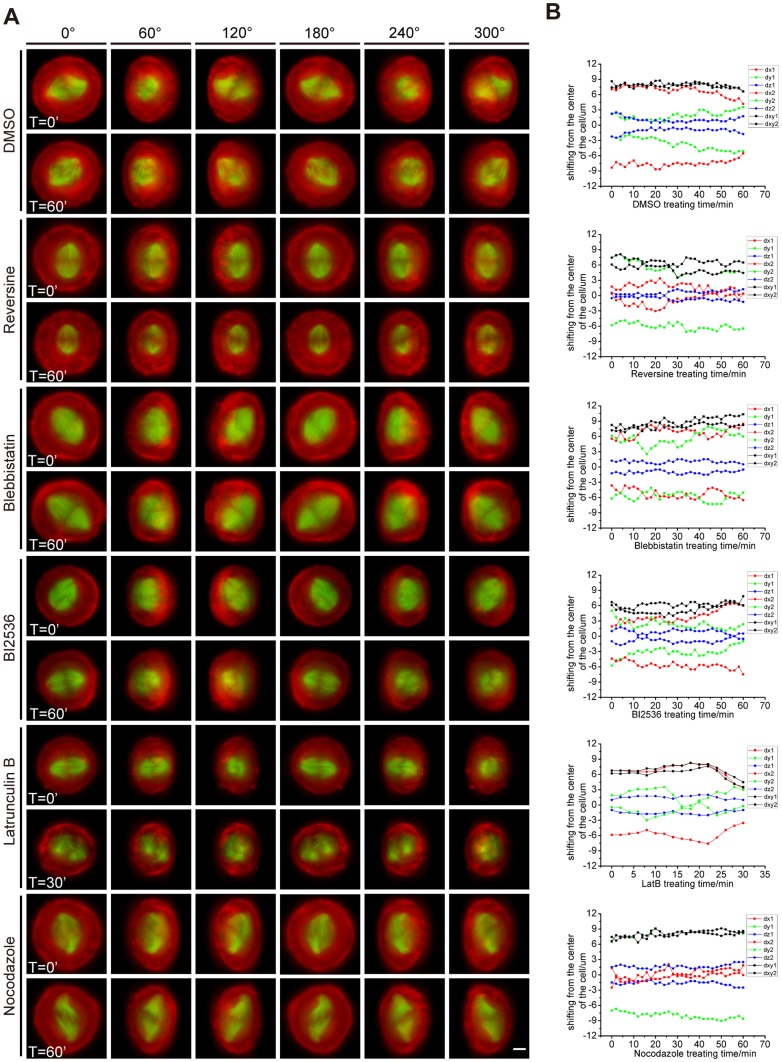
3D projected images of a single HeLa cell and 3D spindle positioning analysis. (A) 3D projected images of cells treated with DMSO, Reversine, Blebbistatin, BI2536, Lat B and Nocodazole. Cells were transfected with mCHerry-UtroCH (red) and GFP-tubulin (green) to label F-actin and microtubule. We added the drugs at time point T = 0' and we ended up taking images at T = 60', respectively. The spindle of cell treated with Lat B deformed critically after T = 30'. The cells are displayed in different rotating angles, 0°, 60°, 120°, 180°, 240° and 300°. Scale bar is 5 µm. (B) The spindle positioning plotting of the 3D projected images along time, and dx, dy and dz represent the coordinate value of spindle pole on X-axis, Y-axis and Z-axis, respectively. Numbers 1 and 2 represent the two spindle poles in each cell (Table S2). Then (dx_1_+dx_2_)/2, (dy_1_+dy_2_)/2 can be used to indicate the position of spindle. Red line represents the shifting on X-axis, green line represents the shifting on Y-axis, blue line represents the shifting on Z axis, and black line stands for the shifting on X-Y section. The rotation of spindle can be calculated between the red and green curves of each spindle pole. Spindle oscillation can be inferred from the vibrating amplitude and frequency of each red or green curve.

To assess the influence that the drugs act on the ring-like F-actin structure and spindle, we acquired multiple-layer living cell images and made 3D projection (Fig. 6A and Movie S8–S19). We expressed mCherry-UtroCH to label F-actin and GFP-tubulin to label spindle. Before imaging, cells were treated with MG132 for 1 hour. Then we added drugs into the medium and labeled this time point as T = 0′. We took each image every 2 minutes, and the overall time was 1 hour. The images were 3D projected and displayed with an interval of 60° from 0° to 300°. We measured the positions of both spindle poles in each cell, and we plotted their shifting from the center of the cell along time (Fig. 6B, Table S2).

Green and red curves represent the position of spindle poles, and the distance between them reflects the length, orientation and position of spindle. Except for the Lat B treated cell, a distinct ring-like F-actin structure can be observed in the projected images. The spindle of DMSO treated cell rotates partially and oscillates with low frequency, and its length changes little along time (Fig. 6B-DMSO). The spindle of Reversine treated cell barely rotates and oscillates (Fig. 6B-Reversine). The spindle of Blebbistatin treated cell rotates slightly but oscillates with high frequency, and it elongates considerably (Fig. 6B-Blebbistatin). The spindle of BI2536 treated cell rotates to some extent and oscillates slightly (Fig. 6B-BI2536). The spindle of Lat B treated cell deforms significantly (Fig. 6B-Lat B). In Nocodazole treated cell, the spindle elongates and deforms, and it oscillates frequently (Fig. 6B-Nocodazole).

We had the 3D overview of the ring-like F-actin structure here. The result of Lat B treatment suggests that the perturbation of the ring-like F-actin structure is coupled with the altered configuration of spindle. The destruction of the structure may lead to uncontrolled spindle geometry rather than spindle length (Fig. S3F). The result of Blebbistatin treatment suggests that Myosin also functions in reducing the frequency of spindle oscillation.

## Discussion

Accurate chromosome segregation in mitosis requires precise coordination of the microtubule-based spindle and the actin-based cell cortex. Thus, the major function of mitotic cytoplasmic actin filament is possible to enable spindle geometry to effectively prevent chromosome instability [38]. Recent studies have suggested a more direct role for the actin cytoskeleton in spindle formation [22], based on observed defects in spindle morphogenesis and orientation following perturbations on mitotic actin cytoskeleton [39], [40], [41], [42] and actin-based hubs such as Myosin 10 and Ezrin-Radixin-Moesin (ERM) family protein [13], [43], [44]. These interactions possibly contribute to centrosome separation and spindle position through Myosin-driven cortical flow. It has been showed that loss of cortical F-actin in mitotic HeLa cells had little impact on overall period of mitosis or spindle bipolarity in isolated culture, even though the cortical F-actin was critically required in mitotic entry [37]. It would be interesting to ascertain the role of cytoplasmic F-actin, especially the ring-like F-actin structure, in mitotic processes such as mitotic entry and spindle bipolarity.

In this study, we analyzed the formation of ring-like F-actin structure using cell biological and computational approaches. The formation of this structure is parallel to spindle positioning and is regulated by Myosin and mitotic kinases, in consistence with the recent literature [13], [29]. To elaborate spindle positioning in a quantitative manner, we formulated a mathematical model to simulate spindle positioning and orientation. According to previous works, we inferred that the cooperation of astral microtubule and cytoplasmic actin filament orchestrates the process of spindle positioning and orientation [2], [3], [10], [36], [37]. Thus, we raised an oscillator model based on previous work and got an approximate solution [35]. We predicted the final spindle pole position with 
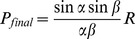
. We also speculated the role of ring-like F-actin structure in spindle positioning with three hypotheses. All three hypotheses result in the decrease of effective connections between astral microtubule and cell cortex. In the model, we neglected the dynamics of kinetochore-microtubule and supposed the spindle is perfect and rigid, regardless of the alteration of spindle plasticity after the addition of these drugs.

However, the utility of our model is limited due to its hypotheses, as the species and inner properties of cells vary. Recent works have focused on how these oscillators act on spindle with pushing and pulling force [35], [36]. In fact, the model we raised has calculated the influence of pulling force produced by the oscillators with D in an interval [D_1_, D_2_] and a positive momentum. However, if we introduce the pushing force produced by the oscillators with D in another interval [D_3_, D_4_] and a negative momentum, the calculation will result in partial neutralization due to the overlapped intervals. Here, [D_3_, D_4_] refers to the situation that the growth of microtubule is more rapid than the velocity that motors walk on microtubule.

We have also raised questions on the formation, the structural property and the physical meanings of the ring-like F-actin structure. We used living cell imaging and immunofluorescence to observe the metaphase arrested HeLa cells treated with different drugs. We found that the inhibitors delay the formation of the ring-like F-actin structure to different extents. Interfering the formation of the ring-like F-actin structure by these inhibitors is accompanied with altered spindle positioning and asymmetric cell division simultaneously. The 3D projection has also suggested possible links between the ring-like F-actin structure and spindle positioning. Further experiments are needed to clarify the molecular mechanism.

It is important to study cytoplasmic F-actin network and screen the molecules participating in the interactions between cytoplasmic actin filament and spindle microtubule. Cytoplasmic F-actin network may function as a potential interactive hub to orchestrate spindle positioning, and some factors such as ERM members will likely come into play [45], [46]. In a word, mechanical analysis and cytoplasmic factors are the center of the following study as well as molecular mechanism and cortical complex. The most intuitive meaning of studying the interaction between cytoplasmic actin filament and spindle microtubules is its contribution to artificially induce the growth of a group of cells. On the other hand, the study on cytoplasmic actin filament provides a method to study proteins involved in mitotic spindle geometry.

## Supporting Information

Figure S1 **The perturbation of ring-like F-actin structure formation by chemical inhibitors and real-time imaging analyses.** Representative images of HeLa cells treated with MG132 plus Reversine(A), Blebbistatin(B), BI2536(C), Lat B(D). MG132 was added at T = −5′, and the other drugs were added at 0′, respectively. We acquired each image every 1 minute, and the overall time was 1 hour. In (A), (B), (C) and (D) cells were transfected with GFP-tubulin (green) and mCherry-UtroCH (red) to label microtubules and F-actin, respectively. Scale bar, 5 µm.
(TIF)

Figure S2 **The perturbation of ring-like F-actin structure formation by chemical inhibitors and immunofluorescence analyses.** Representative immunofluorescence images of HeLa cells treated with MG132 and (A)Reversine, (B)Blebbistatin, (C)BI2536, (D)Lat B. Drug treatment was performed as mentioned in materials and methods, and cells were collected and fixed after drug treatment, respectively. In (A), (B), (C) and (D) cells were fixed and stained with Rhodamine-phalloidin (red), DM1A (green) and DAPI (blue). Scale Bar, 5 µm. The cells in the figures are only examples of each group.
(TIF)

Figure S3 **Treatment of chemical compounds alters symmetric division and related properties of spindle and ring-like F-actin structure.** (A) and (B) Representative immunofluorescence images of the groups on symmetric division(A), spindle orientation and relative intensity of ring-like structure in HeLa cells arrested in metaphase for 2 hours(B). Cells were released after double blocked by Thymidine and arrested in metaphase by MG132. Drug treatment were performed simultaneously and lasted for 2 hours. Except for Lat B group, a clear ring-like F-actin structure forms with the treatment of these drugs. This ring-like F-actin staining was mainly observed in metaphase or anaphase of asynchronized cells, and it was observed in all MG132-synchronized cells. In (A) and (B) cells were stained for F-actin (red), microtubule (green) and DNA (blue). Scale bar, 5 µm. (C) The ring-like F-actin structure is isotropic. We chose the planes parallel to the Z = 0 plane (Fig. 1C) with the interval of 1 µm between each plane, and the maximum interval is 5 µm. We equally divided each plane into 16 sectors and measured the apparent intensity of F-actin in each part of the circle 2, 3, 4 mentioned in Fig. 3. They are labeled as intracellular maximum, intracellular minimum and cell membrane. Then we got 16 values of each sectors on each plane. We compare the difference between Z = 0 plane and the others using t-test and F-test. Most of the p-value indicate that there is no significant difference between the planes, which means that the Z = 0 plane is sufficient for measurement. (D) Relative intensity of the ring-like F-actin structure in metaphase arrested cells (the same cells as displayed in Fig. S3B) treated with MG132 for 2 hours. The p-value of t-test between groups and control are marked. Except for Lat B group (significantly decrease) and Reversine group (no significance), the other groups have a significantly enhanced ring-like F-actin structure. Long red bars represent the mean value, and the short red bars represent the mean value ±3xSD. (E) The kymograph presenting the motion pattern of the ring-like F-actin structure. We select the first image of the movies (Movies B). Scale bar, 5 µm. Then we make kymographs by using a segment parallel (0°) or vertical (90°) to equate plate. Irregular teeth sequence is observed just inside the cortex, and it is different to previously reported [30], [44]. Scale bar, 2.5 µm (horizontal, the length of the segment is 30 µm, relative to cellular scale) and 10 minutes (vertical, the total time of the movie is 40 minutes). Cells were transfected with GFP-H2B and mCherry-UTRO to label chromosomes and F-actin. (F) Spindle lengths in metaphase arrested cells (the same cells as displayed in Fig. S3B) treated with MG132 for 2 hours. The p-value of t-test between groups and control are marked. The spindle length in BI2536 and Nocodazole groups is significantly shorter than the control, and other groups have no significant difference. Long red bars represent the mean value, and the short red bars represent the mean value ±3xSD.
(TIF)

Table S1 **The numerical values of results in figures.** The worksheets titled “Fig. 3D”, “Fig. 4C”, “Fig. 5C and 5D”, “Fig. 5E and 5F” and “Fig. S3D and S3F” present the raw data of values, average, standard deviation or significance in the corresponding figures as additional remarks, respectively. The worksheet titled “isotropic analysis-Fig. S3C” presents the significance between the planes along Z-axis. The quantitative method is illustrated in the legend of Fig. S3C.
(XLS)

Table S2 **The coordinate values of spindle poles in 3D projection.** This table additionally explains the numerical values in Fig. 6B. The worksheets titled “DMSO-MG132”, “BI2536-MG132”, “Reversine-MG132”, “Blebbistatin-MG132”, “Lat B-MG132” and “Nocodazole-MG132” present the directly measured coordinates of spindle poles (X1, X2, Y1, Y2, Z1, Z2) at each time point with corresponding drug treatments. Additionally, the converted coordinates according to the spindle coordinate instructed in Fig. 2B are presented (dx1, dx2, dy1, dy2, dz1, dz2).
(XLS)

Movie S1 **The motion pattern of the ring-like F-actin structure in metaphase arrested Cell A.** We took each image every 1 minute, and the total time was 40 minutes. Scale bar, 10 µm.
(AVI)

Movie S2 **The motion pattern of the ring-like F-actin structure in metaphase arrested Cell B.** We took each image every 1 minute, and the total time was 40 minutes. Scale bar, 10 µm.
(AVI)

Movie S3 **The real time images on the plane Z = 0 of the DMSO-MG132 treated cell.** We took each image every 1 minute, and the total time was 1 hour. Scale bar, 10 µm.
(AVI)

Movie S4 **The real time images on the plane Z = 0 of the Reversine-MG132 treated cell.** We took each image every 1 minute, and the total time was 1 hour. Scale bar, 10 µm.
(AVI)

Movie S5 **The real time images on the plane Z = 0 of the Blebbistatin-MG132 treated cell.** We took each image every 1 minute, and the total time was 1 hour. Scale bar, 10 µm.
(AVI)

Movie S6 **The real time images on the plane Z = 0 of the BI2536-MG132 treated cell.** We took each image every 1 minute, and the total time was 1 hour. Scale bar, 10 µm.
(AVI)

Movie S7 **The real time images on the plane Z = 0 of the Lat B-MG132 treated cell.** We took each image every 1 minute, and the total time was 1 hour. Scale bar, 10 µm.
(AVI)

Movie S8 **The 3D projected images of DMSO treated cell at time point T = 0′.** The structure is observed every 60° from 0° to 360°. We took images every 2 minutes, and the total time was 1 hour. Scale bar, 10 µm.
(AVI)

Movie S9 **The 3D projected images of DMSO treated cell at time point T = 60′.** The structure is observed every 60° from 0° to 360°. We took images every 2 minutes, and the total time was 1 hour. Scale bar, 10 µm.
(AVI)

Movie S10 **The 3D projected images of Reversine treated cell at time point T = 0′.** The structure is observed every 60° from 0° to 360°. We took images every 2 minutes, and the total time was 1 hour. Scale bar, 10 µm.
(AVI)

Movie S11 **The 3D projected images of Reversine treated cell at time point T = 60′.** The structure is observed every 60° from 0° to 360°. We took images every 2 minutes, and the total time was 1 hour. Scale bar, 10 µm.
(AVI)

Movie S12 **The 3D projected images of Blebbistatin treated cell at time point T = 0′.** The structure is observed every 60° from 0° to 360°. We took images every 2 minutes, and the total time was 1 hour. Scale bar, 10 µm.
(AVI)

Movie S13 **The 3D projected images of Blebbistatin treated cell at time point T = 60′.** The structure is observed every 60° from 0° to 360°. We took images every 2 minutes, and the total time was 1 hour. Scale bar, 10 µm.
(AVI)

Movie S14 **The 3D projected images of BI2536 treated cell at time point T = 0′.** The structure is observed every 60° from 0° to 360°. We took images every 2 minutes, and the total time was 1 hour. Scale bar, 10 µm.
(AVI)

Movie S15 **The 3D projected images of BI2536 treated cell at time point T = 60′.** The structure is observed every 60° from 0° to 360°. We took images every 2 minutes, and the total time was 1 hour. Scale bar, 10 µm.
(AVI)

Movie S16 **The 3D projected images of Lat B treated cell at time point T = 0′.** The structure is observed every 60° from 0° to 360°. We took images every 2 minutes, and the total time was 1 hour. Scale bar, 10 µm.
(AVI)

Movie S17 **The 3D projected images of Lat B treated cell at time point T = 30′.** After 30 minutes, the cell was unshaped for measuring. The structure is observed every 60° from 0° to 360°. We took images every 2 minutes, and the total time was 1 hour. Scale bar, 10 µm.
(AVI)

Movie S18 **The 3D projected images of Nocodazole treated cell at time point T = 0′.** The structure is observed every 60° from 0° to 360°. We took images every 2 minutes, and the total time was 1 hour. Scale bar, 10 µm.
(AVI)

Movie S19 **The 3D projected images of Nocodazole treated cell at time point T = 60′.** The structure is observed every 60° from 0° to 360°. We took images every 2 minutes, and the total time was 1 hour. Scale bar, 10 µm; In all movies, cells were transfected with mCherry-UtroCH (red), GFP-tubulin (green) or GFP-H2B (green) to label F-actin, microtubules and chromosomes, respectively.
(AVI)
