# Supplementary figures and images for: Characterization of Ring-Like F-Actin Structure as a Mechanical Partner for Spindle Positioning in Mitosis
Source: PLoS One. 2014 Oct 9;9(10):e102547. doi: 10.1371/journal.pone.0102547 (PMC4191959; doi:10.1371/journal.pone.0102547)

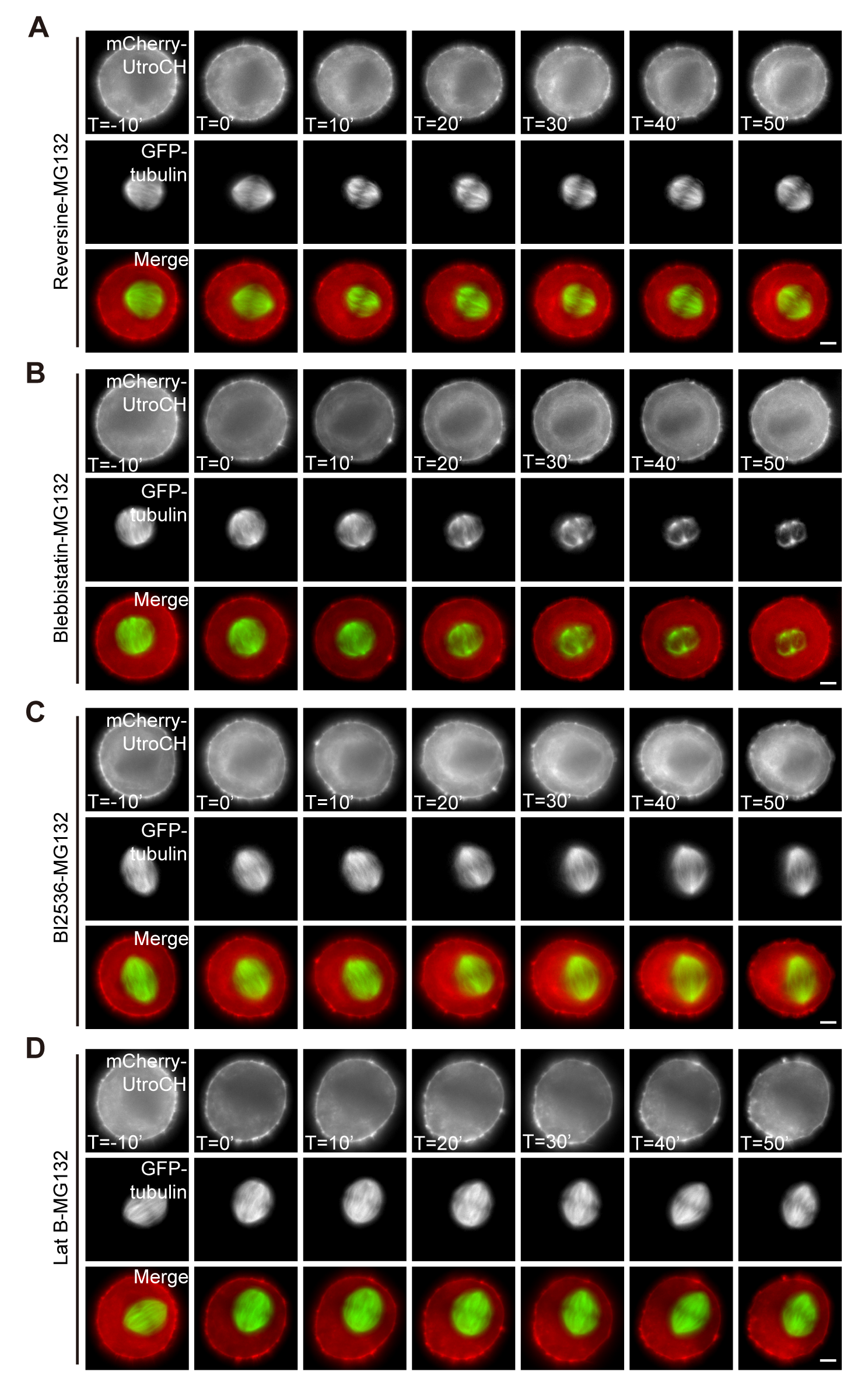

Supplement: Figure S1 — The perturbation of ring-like F-actin structure formation by chemical inhibitors and real-time imaging analyses. Representative images of HeLa cells treated with MG132 plus Reversine(A), Blebbistatin(B), BI2536(C), Lat B(D). MG132 was added at T = −5′, and the other drugs were added at 0′, respectively. We acquired each image every 1 minute, and the overall time was 1 hour. In (A), (B), (C) and (D) cells were transfected with GFP-tubulin (green) and mCherry-UtroCH (red) to label microtubules and F-actin, respectively. Scale bar, 5 µm. (TIF) [file pone.0102547.s001.tif]

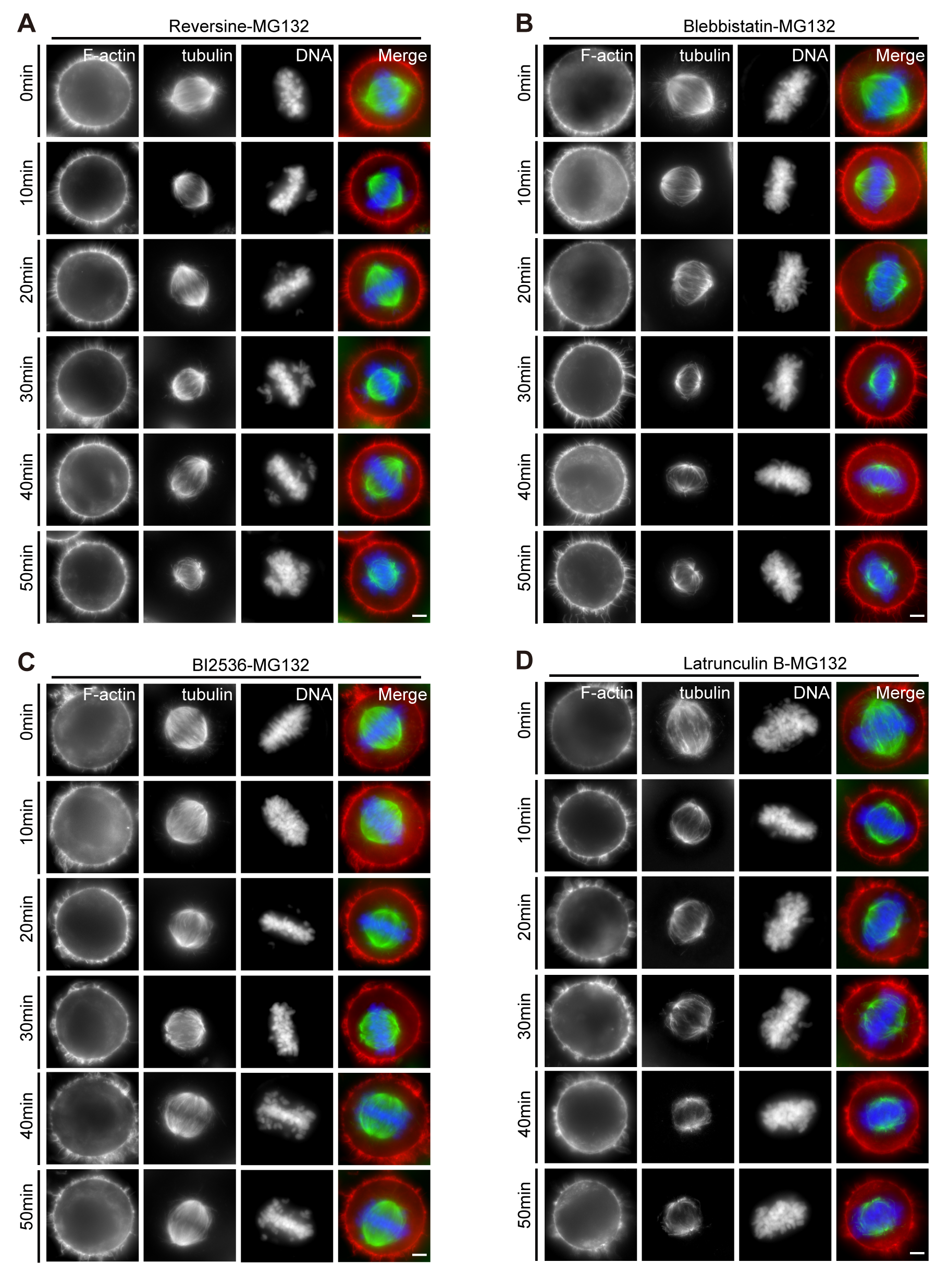

Supplement: Figure S2 — The perturbation of ring-like F-actin structure formation by chemical inhibitors and immunofluorescence analyses. Representative immunofluorescence images of HeLa cells treated with MG132 and (A)Reversine, (B)Blebbistatin, (C)BI2536, (D)Lat B. Drug treatment was performed as mentioned in materials and methods, and cells were collected and fixed after drug treatment, respectively. In (A), (B), (C) and (D) cells were fixed and stained with Rhodamine-phalloidin (red), DM1A (green) and DAPI (blue). Scale Bar, 5 µm. The cells in the figures are only examples of each group. (TIF) [file pone.0102547.s002.tif]

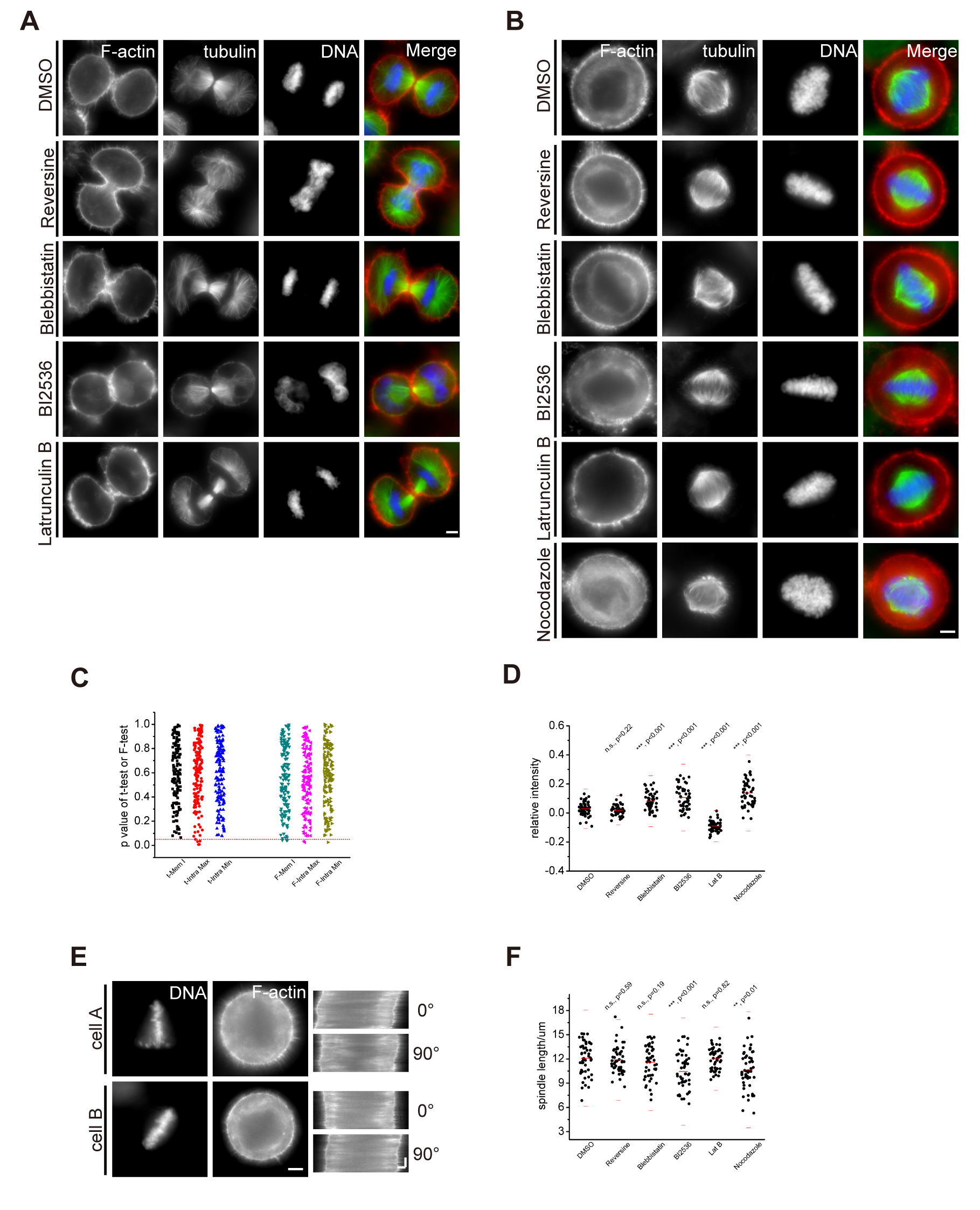

Supplement: Figure S3 — Treatment of chemical compounds alters symmetric division and related properties of spindle and ring-like F-actin structure. (A) and (B) Representative immunofluorescence images of the groups on symmetric division(A), spindle orientation and relative intensity of ring-like structure in HeLa cells arrested in metaphase for 2 hours(B). Cells were released after double blocked by Thymidine and arrested in metaphase by MG132. Drug treatment were performed simultaneously and lasted for 2 hours. Except for Lat B group, a clear ring-like F-actin structure forms with the treatment of these drugs. This ring-like F-actin staining was mainly observed in metaphase or anaphase of asynchronized cells, and it was observed in all MG132-synchronized cells. In (A) and (B) cells were stained for F-actin (red), microtubule (green) and DNA (blue). Scale bar, 5 µm. (C) The ring-like F-actin structure is isotropic. We chose the planes parallel to the Z = 0 plane (Fig. 1C) with the interval of 1 µm between each plane, and the maximum interval is 5 µm. We equally divided each plane into 16 sectors and measured the apparent intensity of F-actin in each part of the circle 2, 3, 4 mentioned in Fig. 3. They are labeled as intracellular maximum, intracellular minimum and cell membrane. Then we got 16 values of each sectors on each plane. We compare the difference between Z = 0 plane and the others using t-test and F-test. Most of the p-value indicate that there is no significant difference between the planes, which means that the Z = 0 plane is sufficient for measurement. (D) Relative intensity of the ring-like F-actin structure in metaphase arrested cells (the same cells as displayed in Fig. S3B) treated with MG132 for 2 hours. The p-value of t-test between groups and control are marked. Except for Lat B group (significantly decrease) and Reversine group (no significance), the other groups have a significantly enhanced ring-like F-actin structure. Long red bars represent the mean value, and the [file pone.0102547.s003.tif]
